# Supplementary material for: Entropy Measures of Electroencephalograms towards the Diagnosis of Psychogenic Non-Epileptic Seizures
Source: Entropy (Basel). 2022 Sep 23;24(10):1348. doi: 10.3390/e24101348 (PMC9601450; doi:10.3390/e24101348)
Supplement: Supplementary file 1 [file entropy-24-01348-s001.zip › entropy-1768323-supplementary.pdf]

Supplementary Materials

Table S1. Balanced accuracies of the approximate entropy of the small subset of data with the test values of m and r_{SD}. Average reports the average accuracy across the band. Ordered from the highest average balanced accuracy to the lowest in the Average column.

m	r_{SD}	**Broad**	**Delta**	**Theta**	**Alpha**	**Beta**	**Gamma**	**Average**
2	0.2	81.81%	**74.40%**	**79.24%**	**81.16%**	72.33%	**68.78%**	**76.29%**
1	0.25	79.77%	73.27%	75.31%	77.39%	74.98%	64.06%	74.13%
2	0.25	81.66%	69.85%	76.43%	75.43%	**75.34%**	60.59%	73.22%
1	0.15	81.66%	73.36%	77.93%	73.40%	70.55%	61.69%	73.10%
1	0.1	**82.30%**	71.99%	76.23%	72.04%	70.34%	63.10%	72.66%
2	0.1	81.49%	68.01%	77.39%	73.14%	70.62%	63.82%	72.41%
2	0.15	80.29%	71.65%	71.51%	73.08%	75.09%	59.03%	71.77%
1	0.2	80.63%	68.59%	74.45%	73.41%	69.78%	59.27%	71.02%

Table S2. Balanced accuracies of the sample entropy of the small subset of data with the test values of m and r_{SD}. Average reports the average accuracy across the band. Ordered from the highest average balanced accuracy to the lowest in the Average column.

m	r_{SD}	**Broad**	**Delta**	**Theta**	**Alpha**	**Beta**	**Gamma**	**Average**
1	0.15	83.34%	**79.16%**	73.28%	**80.65%**	74.75%	68.06%	**76.54%**
1	0.1	**84.14%**	78.34%	77.12%	73.72%	71.91%	65.46%	75.11%
1	0.25	80.18%	77.13%	76.61%	73.83%	72.39%	64.56%	74.12%
2	0.15	79.82%	77.74%	73.81%	69.12%	75.68%	67.38%	73.92%
2	0.25	83.50%	73.07%	**77.90%**	69.06%	65.15%	67.62%	72.72%
2	0.1	80.43%	69.54%	72.86%	65.92%	**75.94%**	**69.08%**	72.30%
1	0.2	78.31%	71.01%	72.65%	70.36%	70.96%	62.74%	71.01%
2	0.2	79.00%	73.29%	71.14%	64.71%	67.31%	64.31%	69.96%

Table S3. Precisions of the entropy metrics for every classifier and EEG frequency band (ECG is included in every band). Bold values denote the highest precision amongst the classifiers for each EEG band and entropy measure.

Features	Classifiers	All	Broad	Delta	Theta	Alpha	Beta	Gamma
Renyi entropy	SVM	93.15%	81.03%	81.45%	83.42%	84.21%	82.41%	82.21%
	kNN	**96.12%**	**88.25%**	**86.04%**	**91.00%**	**91.54%**	**92.63%**	**90.78%**
	RF	92.80%	86.13%	85.15%	89.70%	89.01%	90.32%	89.10%
	GBM	86.14%	78.76%	79.96%	81.73%	81.42%	79.34%	81.28%
Sample entropy $m = 1, r = 0.15$*SD	SVM	87.80%	81.57%	**75.13%**	**77.33%**	**74.17%**	**74.81%**	68.95%
	kNN	**92.08%**	**84.30%**	73.03%	75.21%	70.02%	74.49%	69.16%
	RF	82.82%	82.57%	74.93%	76.23%	70.33%	73.48%	**69.49%**
	GBM	80.77%	77.23%	72.82%	74.75%	71.04%	72.37%	69.35%
Approximate entropy $m = 2, r = 0.2$*SD	SVM	89.00%	82.35%	74.22%	77.95%	**76.65%**	73.53%	69.31%
	kNN	**92.49%**	**85.10%**	72.56%	76.34%	75.67%	**76.42%**	70.04%
	RF	83.42%	83.59%	**74.23%**	**78.35%**	74.66%	74.96%	**70.60%**
	GBM	81.07%	78.11%	72.89%	74.93%	71.84%	72.48%	70.06%
SVD entropy	SVM	87.37%	79.70%	**73.32%**	**74.98%**	**73.05%**	71.03%	**66.86%**
	kNN	**90.49%**	80.21%	68.72%	71.68%	70.42%	70.60%	63.33%
	RF	80.93%	**80.35%**	72.23%	74.03%	71.28%	**72.45%**	64.95%
	GBM	79.97%	76.95%	71.67%	73.47%	69.82%	71.18%	66.17%
Spectral entropy	SVM	84.27%	79.54%	**72.72%**	**73.56%**	**73.63%**	71.93%	67.05%
	kNN	**86.84%**	**80.81%**	70.03%	69.84%	71.57%	73.77%	64.07%
	RF	77.03%	80.35%	72.53%	71.13%	72.30%	**74.41%**	67.04%
	GBM	77.47%	76.78%	71.26%	71.96%	70.41%	72.36%	**68.67%**
Wavelet entropy	SVM	66.65%	**64.99%**	**64.56%**	**71.00%**	61.18%	**61.12%**	60.42%
	kNN	**70.01%**	62.34%	62.54%	66.91%	61.15%	60.15%	59.81%
	RF	63.91%	62.71%	62.10%	67.21%	60.53%	60.81%	60.71%
	GBM	66.55%	62.66%	62.49%	68.78%	**61.41%**	61.00%	**61.77%**

Table S4. Recalls of the entropy metrics for every classifier and EEG frequency band (ECG is included in every band). Bold values denote the highest recall amongst the classifiers for each EEG band and entropy measure.

Features	Classifiers	All	Broad	Delta	Theta	Alpha	Beta	Gamma
Renyi entropy	SVM	93.22%	80.50%	75.62%	87.55%	84.36%	86.84%	83.81%
	kNN	95.19%	83.16%	80.24%	88.79%	89.08%	89.67%	89.46%
	RF	**96.92%**	**88.13%**	**84.65%**	**92.38%**	**92.00%**	**93.59%**	**91.32%**
	GBM	83.70%	72.01%	70.92%	80.44%	81.52%	80.58%	80.77%
Sample entropy $m = 1, r =$ 0.15*SD	SVM	86.46%	65.53%	66.98%	61.53%	58.63%	53.28%	54.39%
	kNN	84.28%	77.00%	66.50%	65.96%	58.68%	65.99%	61.10%
	RF	**87.35%**	**81.74%**	**73.03%**	**74.77%**	**70.87%**	**72.73%**	**71.88%**
	GBM	74.00%	63.36%	62.64%	62.04%	58.74%	57.49%	60.68%
Approximate entropy $m = 2, r =$ 0.2*SD	SVM	87.82%	68.25%	63.26%	62.31%	65.47%	53.74%	52.14%
	kNN	86.28%	76.74%	65.99%	69.09%	66.84%	68.53%	61.88%
	RF	**88.43%**	**81.77%**	**71.95%**	**76.52%**	**76.38%**	**74.34%**	**70.90%**
	GBM	74.92%	64.72%	61.03%	63.15%	63.95%	59.45%	61.47%
SVD entropy	SVM	**85.20%**	62.80%	55.52%	57.33%	58.33%	**74.93%**	53.25%
	kNN	77.01%	71.04%	61.39%	62.30%	61.38%	64.14%	54.66%
	RF	83.24%	**79.01%**	**69.79%**	**69.09%**	**70.93%**	74.79%	**64.82%**
	GBM	71.93%	61.57%	58.82%	57.99%	58.17%	67.93%	52.52%
Spectral entropy	SVM	81.03%	63.06%	59.44%	57.06%	56.99%	74.38%	49.05%
	kNN	71.01%	71.65%	63.57%	60.31%	63.77%	66.04%	54.91%
	RF	**81.25%**	**78.84%**	**72.14%**	**70.85%**	**72.52%**	**76.82%**	**68.14%**
	GBM	69.63%	62.93%	60.72%	58.20%	59.10%	70.30%	53.71%
Wavelet entropy	SVM	68.93%	51.53%	50.48%	55.93%	46.04%	54.90%	51.56%
	kNN	20.51%	53.74%	54.20%	55.01%	51.14%	47.21%	47.07%
	RF	**73.44%**	**65.88%**	**65.19%**	**68.81%**	**63.77%**	**66.74%**	**65.85%**
	GBM	59.26%	49.20%	48.28%	57.85%	45.66%	47.79%	48.56%
